# Adenomatoid tumor of the fallopian tube: A case report and a review of the literature

**DOI:** 10.1016/j.ijscr.2024.110268

**Published:** 2024-09-10

**Authors:** Kyriaki Mitta, Georgios Michos, Eleni Athanasiadou, Evangelos Papanikolaou, Ioannis Tsakiridis, Apostolos Mamopoulos

**Affiliations:** Third Department of Obstetrics and Gynaecology, Aristotle University of Thessaloniki, Greece

**Keywords:** Adenomatoid tumor, Fallopian tube, Salpinx, Histology

## Abstract

**Introduction:**

Adenomatoid tumor is a rare, benign condition, more common in males, that affects the epididymis, spermatic cord and testicular tunics, whereas in females, the uterus and fallopian tubes. This solitary tumor is commonly appearing as an incidental finding. The diverse morphological characteristics of these tumors pose challenges in differential diagnoses.

**Presentation of case:**

We report a case of a 33-year-old woman with a multilocular solid mass identified in a routine gynecological check-up. The cyst was removed laparoscopically, and histology analysis reported an adenomatoid tumor of the fallopian tube. Patient recovered without any additional treatment and within 2 years delivered a healthy offspring.

Extensive literature search was conducted in Pubmed, Embase and Google Scholar for the identification of all relevant case reports or case series of adenomatoid tumors of the fallopian tubes.

**Discussion:**

Literature search revealed other 49 cases of adenomatoid tumors in fallopian tube published. The mean age of patients was 45,6 years (ranged from 29 to 72 years) and the mean diameter of the tumor was 10 mm.

**Conclusion:**

Laparoscopic removal of such a rare, benign tumor is effective and safe.

## Highlights

• Adenomatoid tumor of salpinx is a rare finding.
• Often misdiagnosed.
• Laparoscopic treatment is safe and effective.
• Studies are required to elucidate the pathway connecting inflammation to the development of adenomatoid tumors.

## 1 Introduction

Adenomatoid tumor is a rare, benign neoplasm that is typically found in the genital tract of females and males [1]. It is more common in males, whereas in females, it affects the uterus and fallopian tubes [2]. However, there have been occasional reports of its occurrence in other locations such as the mesentery, mediastinum, adrenal gland, pancreas, heart [3,4].

In women adenomatoid tumors predominantly are found in the fallopian tube, commonly appearing as solitary and incidental findings [2]. The adenomatoid tumor is of mesothelial origin and is the most common benign tumor of the fallopian tube. In cases where they occur in multiples, there is an association with cyclosporine therapy in the context of renal transplantation [5]. The average age of documented cases falls within the range of 35 to 45 years [2]. It manifests as a rounded, unencapsulated mass with firm consistency, resembling a fibroid and displaying a grey or whitish yellow color [6]. Of particular interest to gynecologists is the potential for this tumor to be misinterpreted as borderline or malignant.

Distinctive histological features have led pathologists to recognize it as a distinct entity. The original theory of mesothelial origin by Masson et al. in 1942 was later supported by both light microscopic and ultrastructural studies by Evans et al. and Marcus et al., respectively [7,8]. Recent molecular studies have identified mutations in TRAF7 as defining features of adenomatoid tumors [9]. However, molecular testing is not routinely employed due to their generally recognizable morphology and distinct immunohistochemical profile.

Regarding the macroscopic and microscopic features of the adenomatoid tumors of the fallopian tube, they are well-circumscribed, subserosal, unencapsulated tumors; composed of a single layer of flattened mesothelial cells. Atypia and mitotic activity are usually absent. Smooth muscle fibers may be mixed with the mesothelial proliferation and lymphoid aggregates may also be seen^2^. In some rare cases, infarction may occur, and necrosis may raise the possibility of a malignant process [10]. Adenomatoid tumors have a wide range of morphologies such as glandular, signet ring, solid, cystic or angiomatoid. Concerning the immunohistochemical characteristics, staining supports the mesothelial origin, while there is a positivity in WT-1, pan-keratin, D2–40 and calretinin [11].

In this study, we present a case report of an adenomatoid tumor of the fallopian tube. In addition, an extensive literature review was conducted to summarize the findings of the previously published cases. Increased awareness of this not so common entity may contribute to avoiding potential diagnostic pitfalls.

This case report has been written in accordance with the SCARE criteria [12].

## 2 Presentation of the case

This is a case of a 33-year-old, nulliparous woman who underwent her routine gynecological check-up. Her BMI was 19.4 kg/m^2^ and no past medical or family history reported. A transvaginal ultrasound revealed the presence of an adnexal mass, prompting further investigation. Patient reported no symptoms and physical examination had no findings. Subsequent assessments included an MRI of the lower abdomen, an ultrasound and an IOTA risk evaluation, along with serum cancer marker assessments, to comprehensively investigate this mass.

The magnetic resonance imaging (MRI) of the lower abdomen reported the presence of a cystic mass of undetermined origin, adjacent to the left ovary; its dimensions were 18 × 15 × 20 mm. The T2-weighted images exhibited a high signal intensity, while the T1-weighted images displayed a low signal intensity. Within the cystic mass, a solid element with nodular morphology was identified. Following the intravenous administration of contrast, there was enhancement observed in the cystic wall. No abnormal findings were identified from the uterus and the right adnexa.

**Figs. 1-3:**
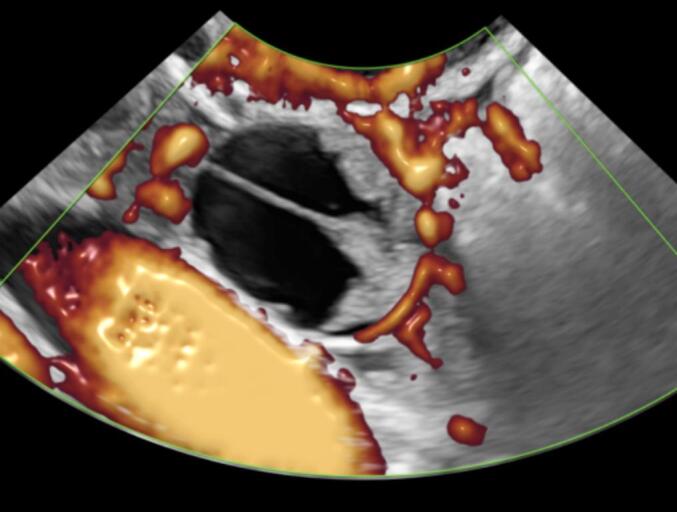

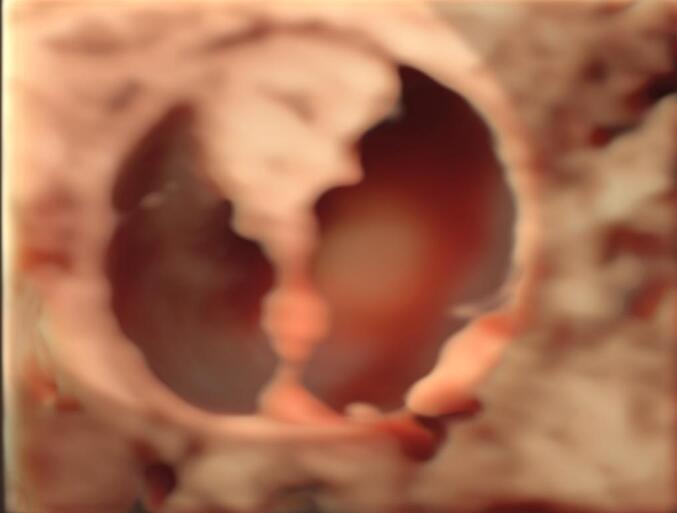

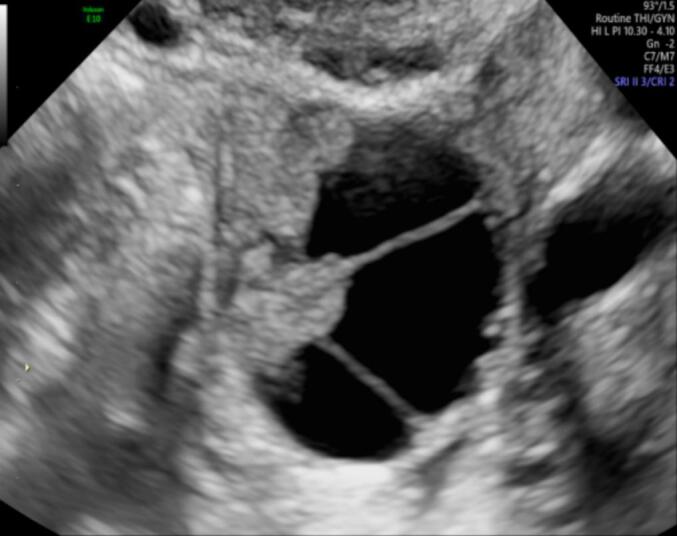
Ultrasonographic appearance of the mass.

The transvaginal ultrasound revealed a multilocular solid mass of undetermined origin situated adjacent to the left ovary. The dimensions of the mass were measured at 20 × 20 × 16 mm. Within the mass, a solid component was identified, with a maximum diameter of 13 mm and height of 7 mm. The dimensions of the uterus were within the normal range, as were those of the right adnexa. The solid component did not exhibit any blood supply in the color Doppler examination. However, it is noteworthy that one of the complete septations within the mass demonstrated evidence of blood supply. The Ca-125 was 13.3 U/ml and all the rest laboratory tests were normal. The International Ovarian Tumor Analysis (IOTA) score was computed employing the ADNEX model, utilizing ultrasonographic findings. The calculated risk for a malignant ovarian tumor was determined to be 9.1 % [13]. Figs. 1-3 show the ultrasonographic appearance of the mass.

**Figs. 4-8:**
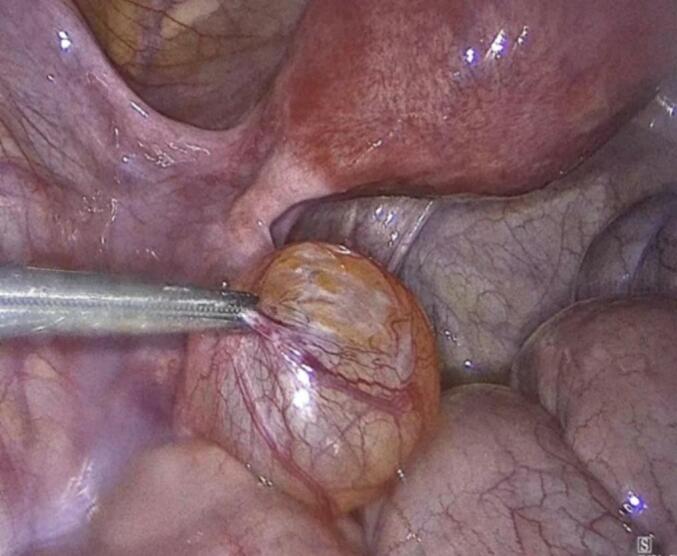

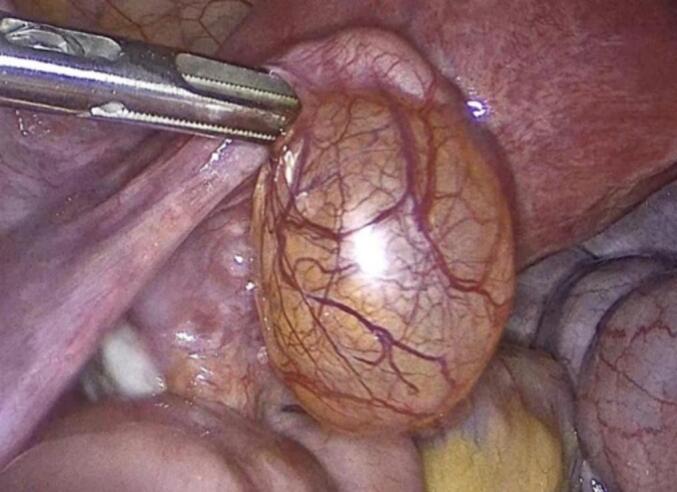

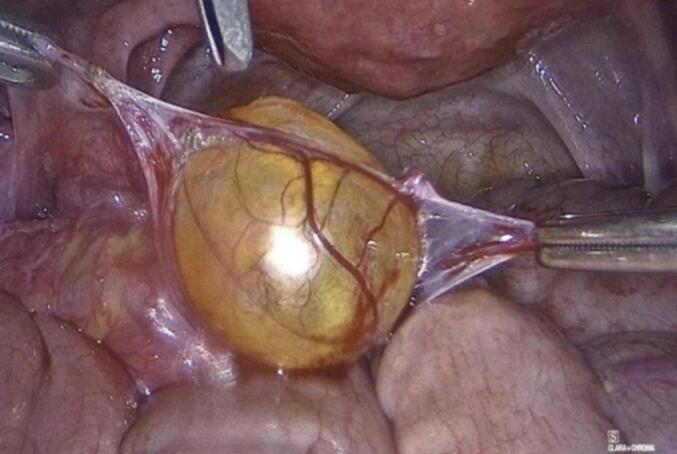

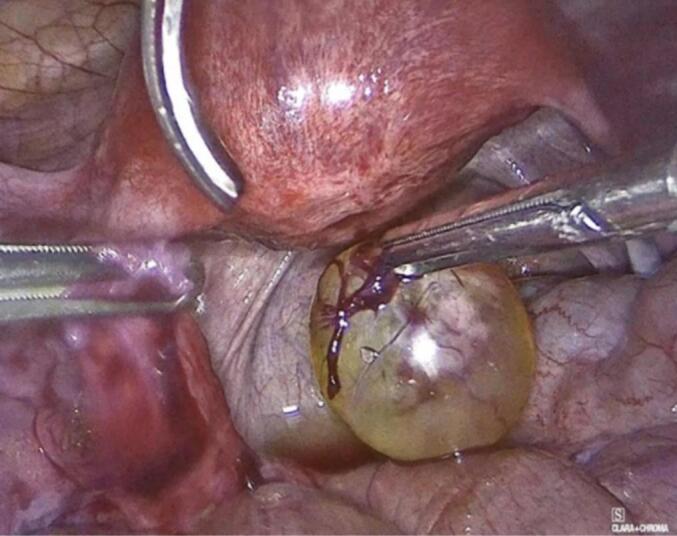

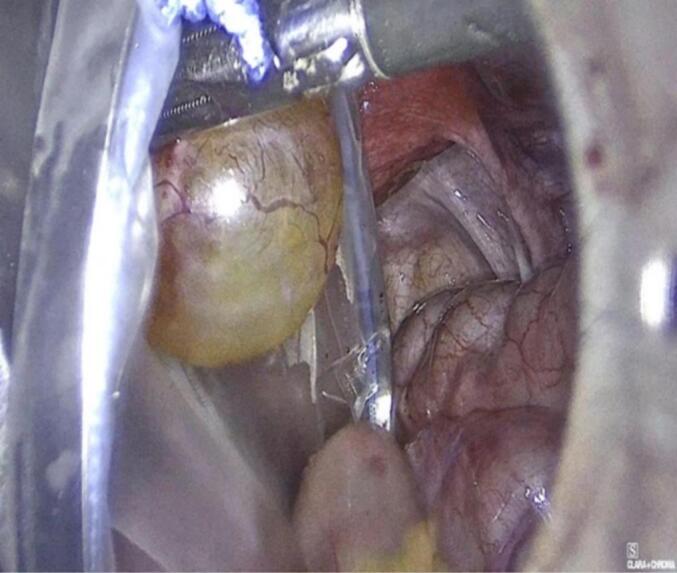
Laparoscopic cystectomy of adenomatoid tumor and in bag extraction.

The patient underwent uncomplicated laparoscopic cystectomy, during which a mass on the left fallopian tube was identified, excised, and subsequently extracted in an endoscopic retrieval bag (Figs. 4-8). The excised mass underwent a rapid biopsy, yielding a negative result for malignancy. Furthermore, random biopsies were obtained from the peritoneum and the pouch of Douglas, and peritoneal washings were collected for cytological examination. Operation performed by a senior consultant, endoscopic surgeon and his team.

The histopathological results showed macroscopically a cystic mass, measuring 17 × 17 mm, with a wall thickness of 1 mm, exhibiting an internal, smooth, whitish-yellow, nodule-like area with a diameter of 10 mm. Paraffin sections confirmed that it is an adenomatoid tumor of the fallopian tube. The formed cystic space presents a cellular mass with slit-like and tubular spaces, covered by a layer of cuboidal, cross-striated cells with scant cytoplasm, without atypia or nuclear abnormalities. The substrate is fibrous. The final diagnosis was an adenomatoid tumor of the fallopian tube, without any evidence of malignancy. The random peritoneal biopsies, along with the cytology of the peritoneal washing were negative for malignancy, as well.

## 3 Discussion

A case of a 33-year-old woman with an adenomatoid tumor of the left fallopian tube was presented. The mass was removed laparoscopically, and the histopathological results confirmed that it was an adenomatoid tumor of the fallopian tube.

The literature review identified 49 cases of adenomatoid tumors in the fallopian tube. The average age at presentation was 45.6 years, with a median size of 1 cm. Recurrence was not documented in any of the reviewed studies. Regarding histopathology, the tumors were predominantly moderately firm, light grey masses without encapsulation. The stroma exhibited mainly fibrovascular features, including muscle bundles and connective tissue. Gland-like spaces were lined by flattened, cuboidal, or columnar cells. Solid nests were commonly formed at the tumor center. Many cases showed focal lymphocyte infiltration, acidophilic cytoplasm, and oval or round nuclei. Adenomatoid tumors were consistently found incidentally in all cases. A study suggested a potential association between mutations in the TRAF7 gene and adenomatoid tumors [9].

Among the cases, 17 had coexisting uterine fibroids. Two cases involved endometrial polyps, one hydrosalpinx, pelvic inflammatory disease, three cases of endometriosis and benign ovarian masses. Other reported coexisting pathologies included cervical cancer, ovarian fibro-thecoma, uterine endometrioid adenocarcinoma, uterine adenomyosis and syphilis. Notably, uterine fibroids were commonly found alongside adenomatoid tumors. Furthermore, literature supports an association between immunosuppression and an increased incidence of adenomatoid tumors [14].

Estimating the precise epidemiological aspects of this tumor is challenging, given that most published cases are either case reports or case series. While the diagnosis is generally straightforward, the diverse morphology presents various potential differential diagnoses.

Differential diagnosis of adenomatoid tumors includes metastatic signet ring cell carcinoma, salpingitis isthmica nodosa, mesothelioma, lipoleiomyoma, and lymphangioma [15]. Metastatic carcinoma involving the fallopian tube can exhibit signet ring morphology, resembling an adenomatoid tumor. Patients with metastatic lesions usually have a known history of prior carcinoma, aiding in recognizing this potential diagnostic pitfall [16]. Although both adenomatoid tumors and carcinomas are positive for keratins, carcinoma is typically negative for calretinin and D2–40, and may be positive for site-specific markers [17].

Salpingitis isthmica nodosa may form a nodular mass with glands resembling an adenomatoid tumor. The glands are lined by tubal-type epithelium, located in the muscular wall of the tube, and have been likened to tubal adenomyosis [18]. Malignant mesothelioma, like adenomatoid tumors, is of mesothelial lineage but forms a large, infiltrative mass with increased cytologic atypia and mitotic activity. In addition to the tubal serosa, other peritoneal surfaces may be involved [19].

Prognosis of adenomatoid tumor is excellent and no further follow up- after the excision is recommended.

## 4 Conclusion

Adenomatoid tumors are rare neoplasms, primarily affecting the uterus, fallopian tubes, and paratesticular tissues. Diagnosis can be challenging. The association with immunosuppression and the oncogenic potential of TRAF7 mutations provide additional insights. Morphological characteristics suggest a connection with well-differentiated papillary mesothelioma and benign multicystic mesothelioma. Additional studies are required to elucidate the pathway connecting inflammation to the development of adenomatoid tumors.

## Patient consent

Written informed consent was obtained from the patient for publication of this case report and accompanying images. A copy of the written consent is available for review by the Editor-in-Chief of this journal on request.

## Ethical approval

This article does not contain any personal information that can lead to the identification of the patient.

As per local policy, Bioethics committee of Medical School, Faculty of Health Sciences, Aristotle University of Thessaloniki, case reports, apart from the informed consent of the patient, does not require ethical approval, as case reports or case series do not constitute research at our institution.

Ethical approval is exempt/waived at our institution for this type of articles.

## Funding

None.

## Author contribution

**Kyriaki Mitta**: Paper design, paper writing.

**Michos George**: Paper design, data collection, paper writing, paper review.

**Eleni Athanasiadou**: Paper design, data collection, picture preparation, paper review.

**Evangelos Papanikolaou**: Paper design, paper review.

**Ioannis Tsakiridis**: Paper design, paper review.

**Apostolos Mamopoulos**: Paper design, data collection, paper review.

## Guarantor

George Michos, Papanikolaou Evaggelos.

## Research registration number

N/A.

## Conflict of interest statement

None.
